# First in human evaluation of [^18^F]PK-209, a PET ligand for the ion channel binding site of NMDA receptors

**DOI:** 10.1186/s13550-018-0424-2

**Published:** 2018-07-27

**Authors:** Jasper van der Aart, Sandeep S. V. Golla, Marieke van der Pluijm, Lothar A. Schwarte, Robert C. Schuit, Pieter J. Klein, Athanasios Metaxas, Albert D. Windhorst, Ronald Boellaard, Adriaan A. Lammertsma, Bart N. M. van Berckel

**Affiliations:** 1 Department of Radiology & Nuclear Medicine, VU University Medical Center, De Boelelaan 1117, 1081 HV Amsterdam, The Netherlands; 2 Centre for Human Drug Research, Leiden, The Netherlands

**Keywords:** PET, NMDA, Glutamate, [^18^F]PK-209, Ketamine

## Abstract

**Background:**

Efforts to develop suitable positron emission tomography (PET) tracers for the ion channel site of human *N*-methyl-d-aspartate (NMDA) receptors have had limited success. [^18^F]PK-209 is a GMOM derivative that binds to the intrachannel phencyclidine site with high affinity and selectivity. Primate PET studies have shown that the volume of distribution in the brain was reduced by administration of the NMDA receptor antagonist MK-801, consistent with substantial specific binding. The purpose of the present study was to evaluate [^18^F]PK-209 in 10 healthy humans by assessing test–retest reproducibility and binding specificity following intravenous *S*-ketamine administration (0.5 mg ∙ kg^−1^). Five healthy subjects underwent a test–retest protocol, and five others a baseline-ketamine protocol. In all cases dynamic, 120-min PET scans were acquired together with metabolite-corrected arterial plasma input functions. Additional input functions were tested based on within-subject and population-average parent fractions.

**Results:**

Best fits of the brain time-activity curves were obtained using an irreversible two-tissue compartment model with additional blood volume parameter. Mean test–retest variability of the net rate of influx *K*_*i*_ varied between 7 and 24% depending on the input function. There were no consistent changes in [^18^F]PK-209 PET parameters following ketamine administration, which may be a consequence of the complex endogenous ligand processes that affect channel gating.

**Conclusions:**

The molecular interaction between [^18^F]PK-209 and the binding site within the NMDA receptor ion channel is insufficiently reproducible and specific to be a reliable imaging agent for its quantification.

**Trial registration:**

EudraCT 2014-001735-36. Registered 28 April 2014

## Background

Ionotropic glutamate receptors are subdivided into four groups: kainate, α-amino-3-hydroxy-5-methyl-4-isoxazolepropionate (AMPA), δ-receptors, and *N*-methyl-d-aspartate receptors (NMDA-R). The NMDA-R is a heteromultimeric assembly of four subunits which, in the central nervous system, is primarily comprised of GluN1 and GluN2(A-D) subunits [1]. The NMDA-R is different from other types of ligand-gated ionotropic receptors in that it functions as a molecular coincidence detector. To open the ion channel, relief of the voltage-dependent magnesium block is required by activation of nearby AMPA and kainate receptors, as well as co-activation of the ligand-binding domain by glutamate (via GluN2) and either d-serine or glycine (via GluN1 or GluN3) [2, 3]. The phencyclidine (PCP) binding site sits within the ion channel in the transmembrane domain of the receptor [4]. It is an attractive target for pharmacological restoration of glutamatergic homeostasis in central nervous system disorders [1]. For example, non-competitive NMDA-R channel blockers such as ketamine, MK-801, and memantine may attenuate excessive neuronal depolarization and prevent amyloid-β-induced excitotoxic cell death [5].

Considerable interest has arisen in the development of a molecular imaging tool that can assess NMDA-R function in vivo. Positron emission tomography (PET) studies of radiolabelled NMDA-R antagonists in humans have been hampered by poor radiotracer selectivity and affinity, low brain entrance, rapid radioligand metabolism, and/or inability to establish specific NMDA-R targeting [6–9]. However, recent studies using [^11^C]GMOM in humans demonstrated that intravenous administration of ketamine 0.3 mg · kg^−1^ reduced the radiotracer net influx rate (*K*_*i*_) in whole brain gray matter by, on average, 66% [10]. The structure of GMOM [11, 12] was used as a template for a new series of substituted *N*,*N*′-diaryl-*N*-methylguanidines [13, 14]. Preclinical experiments showed that the fluorine-18 labeled analogue of GMOM, [^18^F]PK-209 ([3-(2-chloro-5-(methylthio)phenyl)-1-(3-([^18^F]fluoromethoxy)phenyl)-1-methylguanidine]), was the most promising candidate ligand for imaging NMDA-R, with a high apparent affinity of 18 nM against [^3^H]MK-801 (*K*_*i*_) compared with 15 to 22 nM for [^11^C]GMOM [13]. Lipophilicity of [^18^F]PK-209 (LogD_oct,7.4_ = 1.45) was acceptable for a neuroreceptor tracer [13], and the compound exhibited high selectivity for the ion channel of NMDA-R over other targets [15]. A subsequent PET study in rhesus monkeys showed prolonged retention of [^18^F]PK-209 in NMDA-R-rich cortical regions relative to the cerebellum [15]. IV administration of 0.3 mg ∙ kg^−1^ MK-801 30 min before [^18^F]PK-209 PET reduced the volume of distribution (*V*_T_) compared with baseline in two out of three subjects. These findings supported further evaluation of [^18^F]PK-209 as a PET radioligand for the PCP site of the NMDA-R in human subjects. To this end, test–retest reproducibility and specific binding following intravenous administration of ketamine were assessed in this first in human study.

## Methods

### Subjects

This was an open-label study in 10 healthy volunteers aged 22 to 37 years (Table 1). Mean age ± standard deviation (SD) in the test–retest group was 27.6 ± 7.4 and in the ketamine group 24.4 ± 2.2. All subjects were free of medical and psychiatric illnesses based on medical history, neurological examination, blood tests (complete blood count and serum chemistry), urine analysis, and tested negative for drugs in urine.

**Table 1:** Subject demographics and injectate details

	Subject	Gender	Weight (kg)	Age	Scan interval (days)	Injected activity PET1 (MBq)	Injected activity PET2 (MBq)	A_m_ PET1 (MBq · nmol^−1^)	A_m_ PET2 (MBq · nmol^−1^)	Mass PET1 (μg)	Mass PET2 (μg)
Test–retest group	1	F	70	37	47	191	185	41	87	1.6	0.8
	2	M	86	23	35	185	176	87	123	0.8	0.5
	3	M	62	23	28	187	185	88	77	0.7	0.8
	4	F	71	34	61	180	183	45	50	1.4	1.3
	5	M	93	22	31	173	172	60	64	1.0	1.0
	Mean					183	180	64	80	1.1	0.9
	SD					7	6	22	28	0.4	0.3
	Paired *t* test (*P* value)					0.23		0.21		0.22	
Ketamine group	6	F	64	23	161	191	194	47	17	1.4	4.1
	7	M	83	28	28	189	187	89	44	0.8	1.5
	8	F	56	23	98	187	188	80	38	0.8	1.7
	9	M	81	23	49	202	195	52	56	1.4	1.2
	10	F	70	25	13	189	195	10	40	6.4	1.7
	Mean					192	192	56	39	2.2	2.1
	SD					6	4	31	14	2.4	1.1
	Paired *t* test (*P* value)					0.92		0.31		0.94	

*A*_*m*_ molar activity, *SD* standard deviation

### Radiopharmaceutical preparation

Precursor synthesis and radiolabelling procedures for [^18^F]PK-209 have been described previously [13]. The radiotracer was formulated in a phosphate-buffered saline solution containing 6.7% ethanol and administered intravenously as a 0.5 to 15 mL bolus injection, which subsequently was flushed with a saline solution. The molar activity at time of injection was calculated against a calibration curve of PK-209 using the same high-performance liquid chromatography (HPLC) system and was 10–123 MBq · nmol^-^^1^. In all cases, synthesis time, including HPLC purification, was approximately 90 min, and radiochemical purity of the final product > 98%.

### Positron emission tomography data acquisition

In total, 20 PET scans were acquired on a Gemini TF-64 PET/CT scanner (Philips Medical Systems, Cleveland, OH, USA). Five subjects underwent [^18^F]PK-209 test–retest (TRT) scans 31–61 days apart over a period of 211 days. Another five subjects underwent baseline and ketamine scans 13–161 days apart over a period of 258 days. At the start of each scan [^18^F]PK-209 was injected as an intravenous bolus with a mean injected activity of 187 ± 7 MBq. All scans were performed between 12:00 and 15:00 h to minimize diurnal variation. Duration of daylight on each scan day was calculated to investigate seasonal changes in tracer binding. Dynamic PET data were acquired for 120 min and binned into 25 time frames (1 × 15, 3 × 5, 3 × 10, 4 × 60, 2 × 150, 2 × 300, 10 × 600 s). PET was followed by a low-dose CT (30 mAs, 120 kVp) for attenuation correction and localization. Data were reconstructed using a three-dimensional row action maximum likelihood reconstruction algorithm (3-D RAMLA). Acquired PET data were normalized and corrected for dead time, randoms, scatter, attenuation, and decay [16].

### Blood sampling

All subjects received a radial artery cannula for continuous and manual arterial sampling. For each scan, an arterial input function was obtained using an on-line blood sampler (Veenstra Instruments, Joure, The Netherlands) with a withdrawal rate of 5 mL · min^−1^ during the first 5 min and 2.5 mL · min^−1^ from 5 to 60 min post injection (p.i.) in order to stay within blood volume sampling constraints. Continuous blood withdrawal was interrupted briefly to manually collect arterial blood samples (10 mL each) at set timepoints (5, 10, 20, 40, 60, 75, 90, 120 min p.i.). In several subjects, an arterial sample was taken immediately before PET and incubated with [^18^F]PK-209 at 37 °C to compare radioactivity recovery fractions throughout the 120 min with extracted fractions of the arterial samples taken during PET. The samples were used to measure blood and plasma radioactivity concentrations, whole blood-to-plasma ratios, and plasma metabolite fractions of [^18^F]PK-209. For the latter purpose, samples were analyzed using solid-phase extraction combined with HPLC using off-line radioactivity detection.

The final arterial plasma input function was derived using both continuous and discrete arterial blood data. The input function was corrected for plasma to whole blood ratio, metabolites, and time delay. For each scan, two additional plasma input functions were generated to investigate the effect of within-subject and between-subject variability in plasma data. For the within-subject averaged input function, the mean of PET1 and PET2 [^18^F]PK-209 parent fractions per subject were used at each of the eight sample timepoints for both scans. For the population-averaged input function, the mean parent fractions for all five subjects in the TRT and ketamine groups (i.e., 10 scans) were taken at each timepoint. The manual arterial blood samples were also used for assessment of the plasma ketamine concentration. The area under the ketamine concentration–time curve was estimated by means of the trapezoidal rule.

### Ketamine infusion

The (S+)-isomer of ketamine (0.5 mg · kg^−1^) was dissolved in NaCl 50 mL and administered intravenously in a pseudo-steady state model with a sub-acute loading dose. First, ketamine was administered for 40 min at a rate of 0.006 mg · kg^−1^ · min^−1^ followed by a 130 min equilibrium phase at 0.002 mg · kg^−1^ · min^−1^. [^18^F]PK-209 injection and start of PET were performed at 10 min into the equilibrium phase, following 0.26 mg · kg^−1^ administration in the preceding 50 min.

### Image acquisition and analysis

Prior to the PET studies, structural three-dimensional coronal T1-weighted magnetic resonance images (MRI) were acquired for all subjects on a 1.5T Sonata scanner (Siemens Medical Solutions, Erlangen, Germany) with 160 coronal slices using echo time 3.97 ms, repetition time 2700 ms, flip angle 8°, and voxel size 1.0 × 1.5 × 1.0 mm^3^. Subject motion was checked by visual inspection of the alignment of intrasubject PET frames, and no frames were excluded due to excessive movement. T1-weighted MRI scans were co-registered to an average of the PET frames from ~ 4–70 min p.i. using VINCI software [17]. The co-registered MRI scans were segmented into gray matter (GM), white matter, and extra-cerebral fluid, using PVE-lab [18]. Subsequently, 68 regions of interest (ROIs), including whole brain, were derived from the Hammers atlas [19] and used to extract GM time-activity curves (TACs). As a last step, the TACs of 60 out of the 67 ROIs were redefined into nine volume-weighted ROIs by combining the radioactivity at each timepoint according to the equation $\overline{Bq}=\frac{\sum \limits_i{\alpha}_i\times {\mathrm{Bq}}_i}{\sum \limits_i{\alpha}_i}$, where *α*_*i*_ is the volume (in mm^3^) of region *i*, and Bq_*i*_ the counts per mm^3^ of region *i*. The selection of ROIs was based on the widespread NMDA-R availability in the brain: frontal, temporal, occipital, and parietal cortices; hippocampus; thalamus; insula; brainstem; dorsal striatum; and cerebellum, as well as whole brain GM from the Hammers atlas. Standardized uptake values (SUV; equalling measured activity divided by injected dose per body weight) were calculated for whole brain GM.

Using the metabolite-corrected arterial plasma input function, all GM TACs were fitted to single-tissue (1T2k), two-tissue irreversible (2T3k), and reversible (2T4k) compartmental models, both with and without an additional parameter for fractional blood volume (*V*_B_). The non-metabolite-corrected arterial whole blood curve was used as input function for *V*_B_. The optimal tracer kinetic model for describing in vivo kinetics of [^18^F]PK-209 was determined according to the Akaike Information Criterion (AIC) [20]. Depending on the model preference, outcome measures were the net influx constant (*K*_*i*_ = *K*_1_ *k*_3_/(*k*_2_ + *k*_3_)) or the distribution volume $\left({V}_T=\frac{K_1}{k_2}\left(1+\frac{k_3}{k_4}\right)\right)$. For subsequent group level analysis (e.g., TRT variability and ketamine effects), all scans were analyzed using the same, most preferred, tracer kinetic model. Non-displaceable distribution volumes (*V*_ND_ = *K*_1_/*k*_2_) as well as *K*_1_ and *k*_3_ values are also reported separately.

### Statistics

The absolute percentage TRT variability of kinetic parameters between test and retest scans was calculated using the equation TRT variability = 2 · (ρ_retest_ − ρ_test_)/(ρ_test_ + ρ_retest_) · 100, where *ρ* is the kinetic parameter of interest. The between-subject coefficient of variation was calculated as the standard deviation divided by the sample mean · 100. Areas under the curve (AUC) from 0 to 120 min (SUV_AUC_) and plasma parent fractions (0–180 s and 0–120 min) were calculated with GraphPad Prism 7.0 (La Jolla, CA, USA, www.graphpad.com). SUV from 90 to 120 min (SUV_90–120_) was calculated as the mean of the last three mid-frame SUV values.

## Results

### Brain images and plasma analysis

**Fig. 1:**
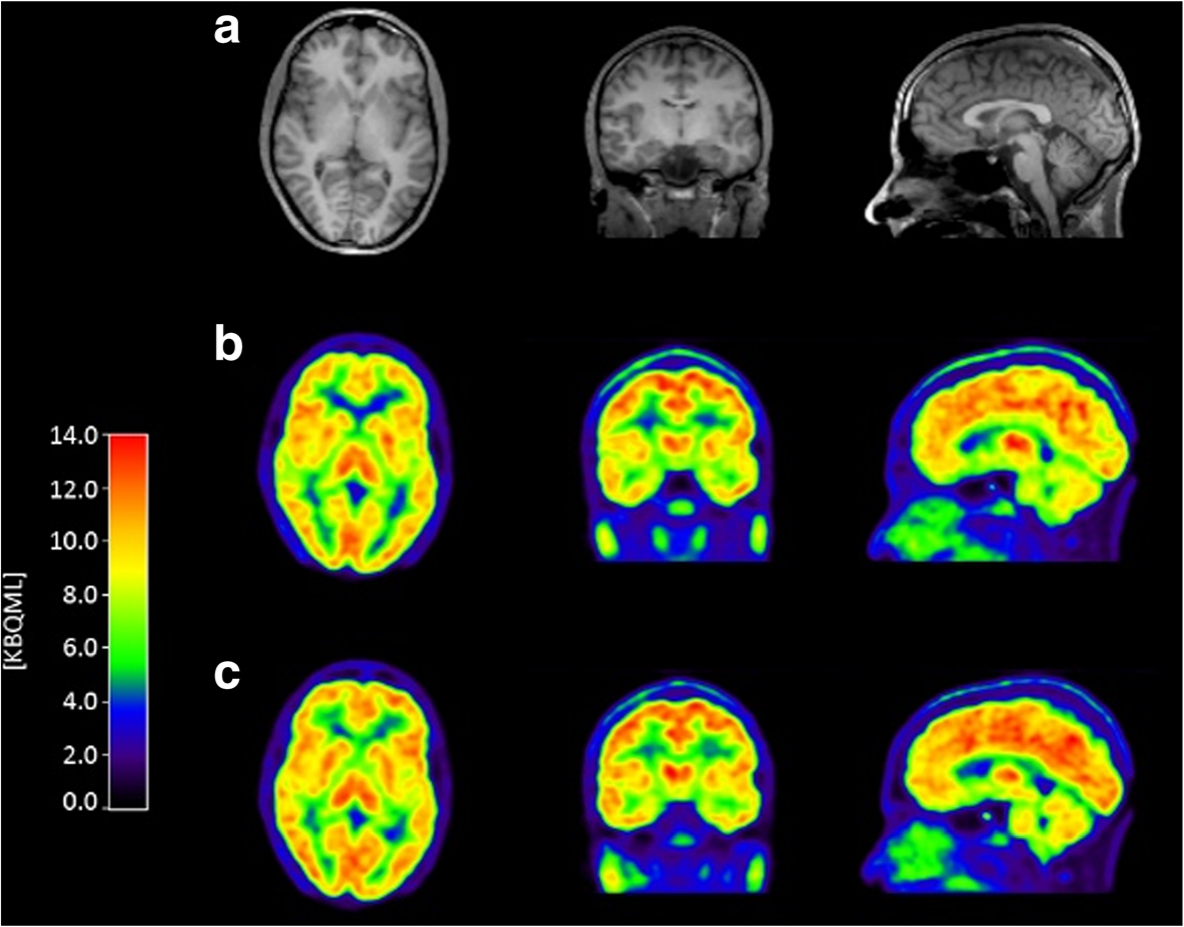
Transaxial, coronal, and sagittal views of MR images (**a**) and averaged PET images from 4.25–70 min for the test scan (**b**) and retest scan (**c**) of subject 1. Activity is expressed in kBq · mL^−1^

**Fig. 2:**
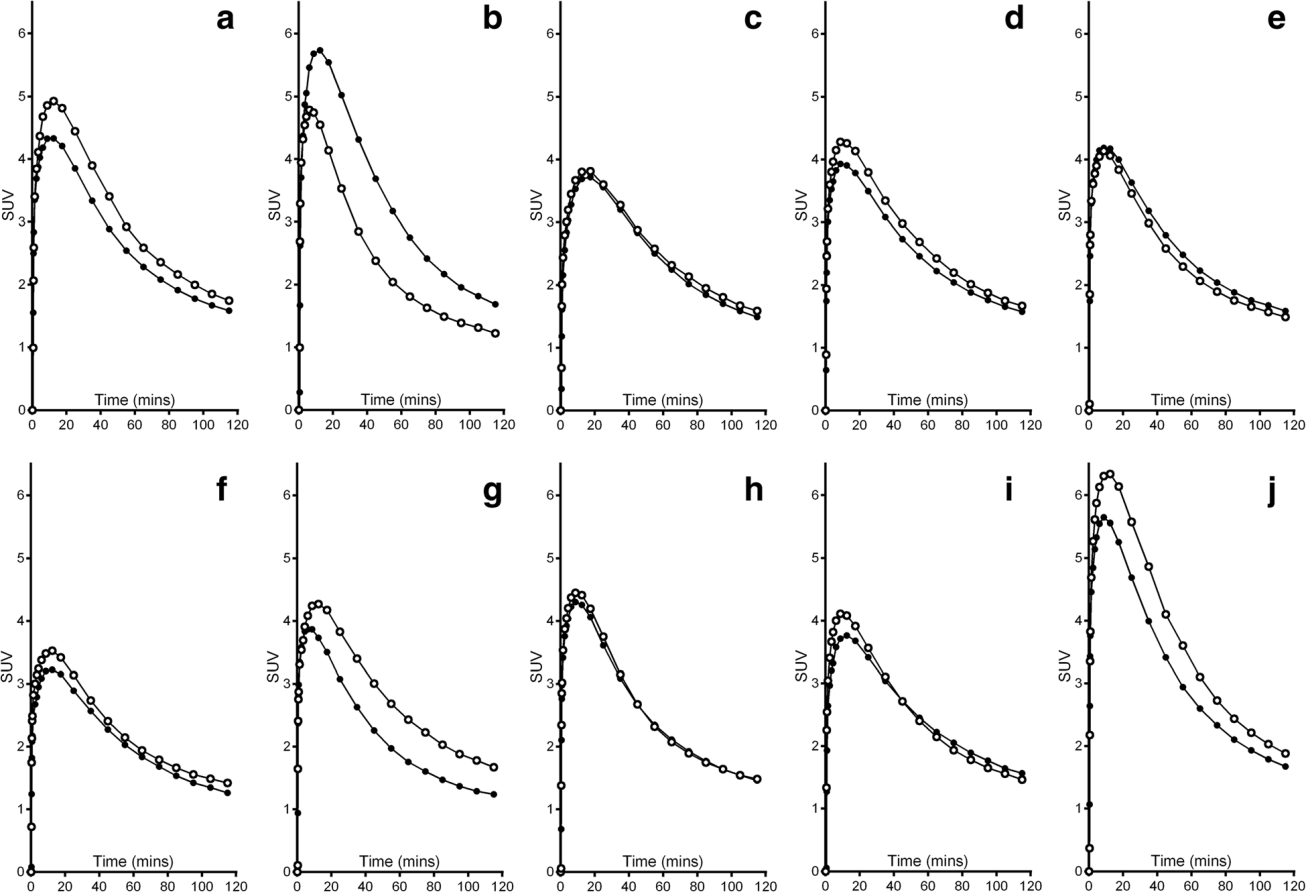
Time-activity curves for all subjects. **a**–**e** Test–retest scans (closed and open circles respectively) of subjects 1 to 5. **f**–**j** Baseline and ketamine scans (closed and open circles respectively) for subjects 6–10

**Fig. 3:**
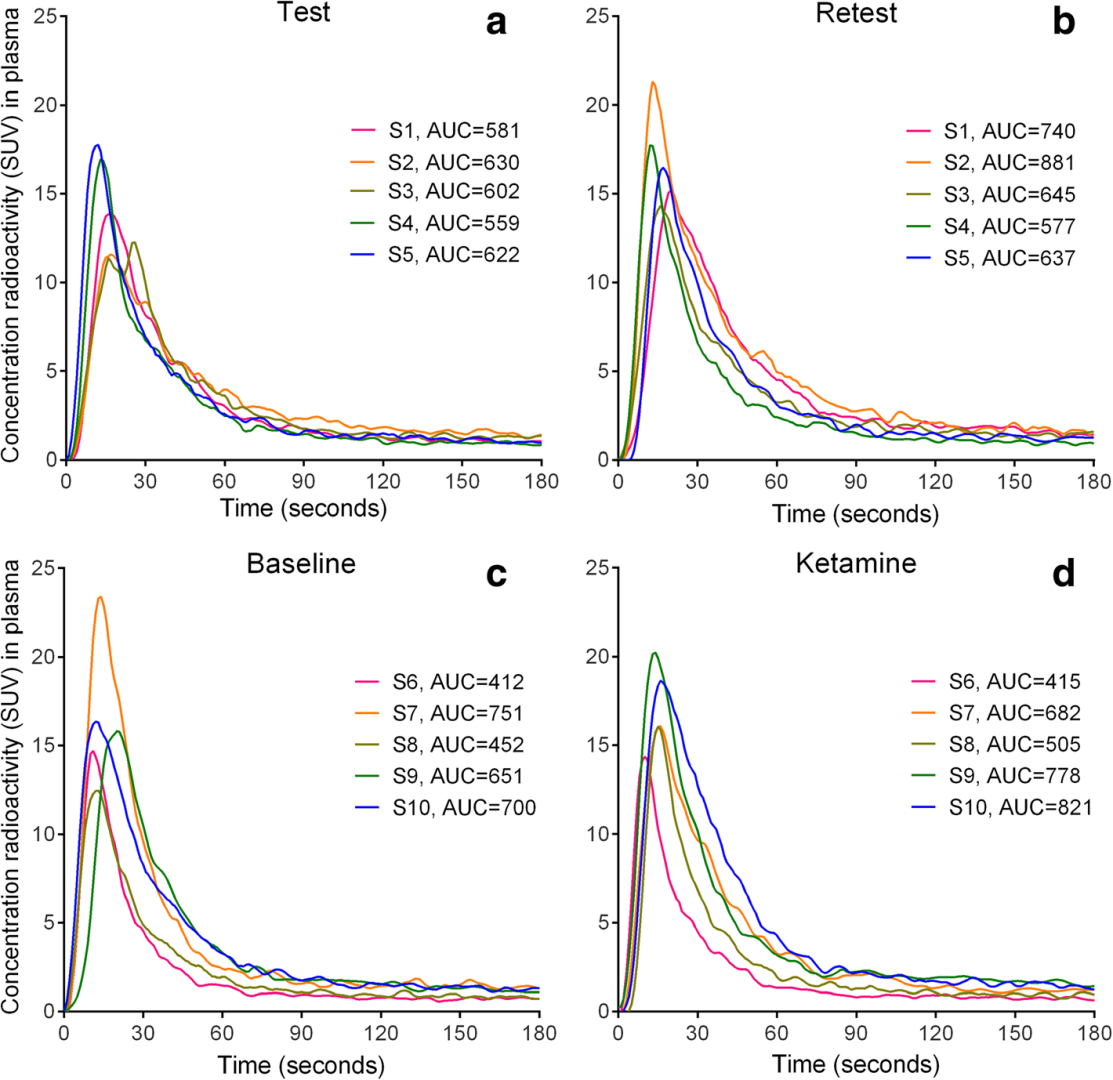
Concentration of total radioactivity in plasma following [^18^F]PK-209 injection for subject 1–5 test scans (**a**) and retest scans (**b**) and subject 6–10 baseline scans (**c**) and ketamine scans (**d**)

**Fig. 4:**
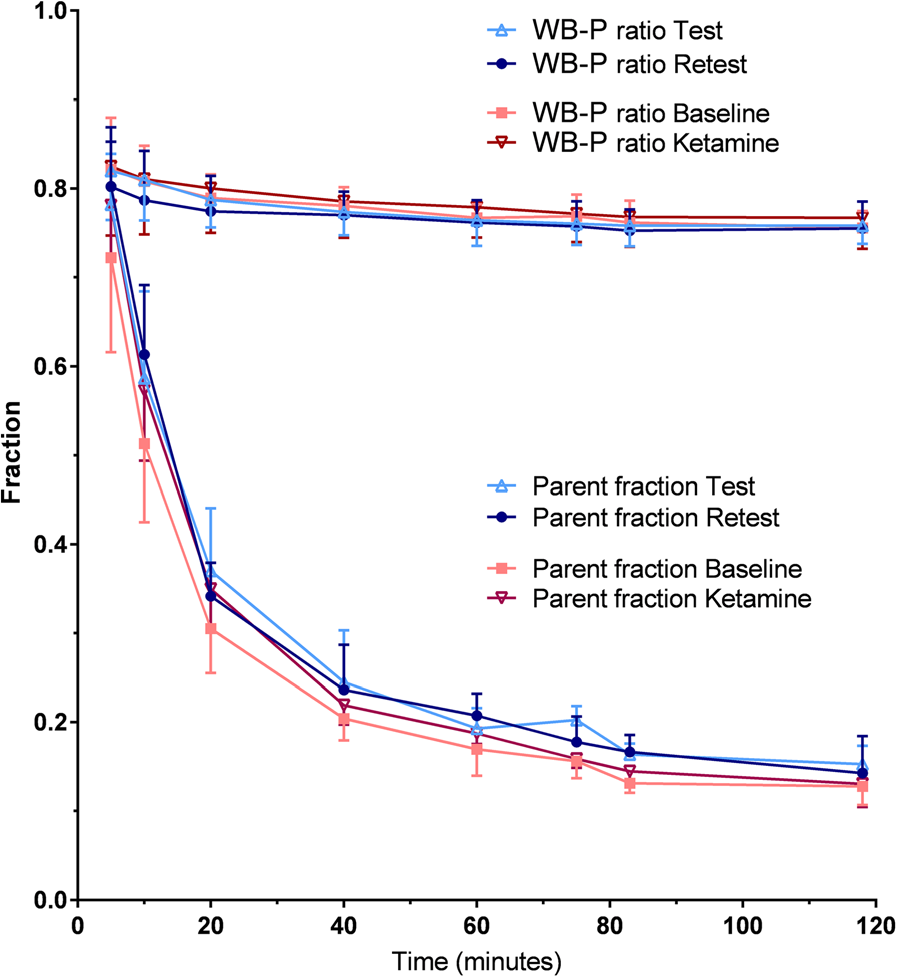
Mean [^18^F]PK-209 parent fractions in plasma and whole blood-to-plasma (WB-P) ratios of the five subjects in each group

Uptake images from a TRT subject are shown in Fig. 1. Total GM TACs for all subjects are shown in Fig. 2. Peak uptake occurred around 10 min p.i. and with a SUV between 3.6 and 5.7. Brain activity decreased to about 25–40% of the peak by 120 min. No radioactivity uptake was observed in the jaw and skull in the dynamic PET images, indicating no defluorination. Injected activity was similar between PET1 and PET2 in the TRT (subjects 1–5) and ketamine (subjects 6–10) groups (Table 1). Molar activity and injected mass differed on average (± SD) 50 ± 40% between PET1 and PET2. The radioactivity concentration of [^18^F]PK-209 in plasma peaked with a mean SUV of 17.4 ± 2.8 and decreased to ~ 7% of the peak at 3 min (Fig. 3). The AUC (0–180 s) for both TRT and ketamine groups did not differ between PET1 and PET2 (paired Student’s *t* test *p* = 0.11 and *p* = 0.20, respectively). [^18^F]PK-209 was rapidly metabolized (Fig. 4). Mean parent fractions for all 20 scans were 77 ± 7, 57 ± 9, 34 ± 5, 19 ± 3, and 14 ± 3% at 5, 10, 20, 60, and 120 min p.i., respectively. HPLC analysis of the radioactivity in plasma demonstrated that one major metabolite accounted for 8 ± 5% at 5 min, 27 ± 3% at 20 min, and 32 ± 3% at 120 min p.i. The remainder of the activity could not be extracted, constituting 15 ± 3% at 5 min and 54 ± 4% at 120 min p.i. Recovery from the incubated pre-PET arterial sample remained > 90%. There was no significant difference in metabolism (AUC) of [^18^F]PK-209 between test and retest scans. However, parent fractions in the baseline scans of subjects 6 to 10 were 8 to 15% lower than their ketamine scans and 16% lower (range − 5 to − 30%) than the test–retest group. Specifically, the parent fraction was significantly lower at 10, 20, and 80 min post [^18^F]PK-209 injection (paired Student’s *t* test *p* < 0.05). The difference in mean blood-to-plasma ratios between test–retest and between baseline–ketamine scans was < 3% for all eight measured timepoints.

### Kinetic modeling

Best model fits, according to the AIC, were obtained using an irreversible two-tissue compartment model with blood volume parameter (2T3k_V_B_) in 8 out of the 10 TRT scans and 6 out of the 10 ketamine group scans (see Table 2). Model preference between baseline and ketamine scans was different in four out of five subjects, but no consistent change in model preference was observed between pre- and post-ketamine scans. Fits of the 67 ROIs to the 2T3k_V_B_ model in the 15 non-ketamine PET scans showed the highest consistency in the temporal lobe and cerebellum (12 out of 15 scans preferring 2T3k_V_B_) and the lowest consistency in the thalamus and brainstem (7 and 8 out of 15 scans, respectively). The correlation between model agreement and ROI size was significant (*r* = 0.33, *p* < 0.01) indicating less stable fits in smaller regions. The 2T4k_V_B_ model was preferred in 25% of the ROIs, and no regions were identified that consistently favored a model other than the 2T3k_V_B_ model. Input functions that were averaged within-subjects increased the consistency in model preference, as 17 out of 20 PET scans showed best fits for the 2T3k_V_B_ model. Considering the finding that the majority of scans as well as ROIs were fit best by the irreversible two-tissue compartment model, all scans were analyzed using the 2T3k_V_B_ model. The primary outcome parameter was therefore the net influx rate constant. *K*_*i*_ values for all 20 scans were calculated with three different plasma input functions and are presented in Table 2. The coefficient of variation of the baseline (PET1) *K*_*i*_ values (*N* = 10) was 36% using the single scan input function, 37% using the within-subject input function, and 43% using the population average input function. Mean PET1 values of *K*_*i*_ in the nine volume-weighted ROIs, from low to high, were similar for all three input functions at approximately 0.008 in dorsal striatum; 0.010 in cerebellum, thalamus, and insula; 0.015–0.018 in frontal, temporal, parietal, and occipital cortices; and 0.034–0.040 in brainstem. Correlations between *K*_*i*_ and SUV_90–120_ of the 10 PET1 scans and between Δ*K*_*i*_ and ΔSUV_90–120_ were low for all three input functions (Spearman’s *ρ* < 0.42, *p* > 0.22).

**Table 2:** Rate constants obtained using a 2T3k_V_B_ model and three different plasma input functions (IF) for all subjects

		Single scan IF						Within-subject IF			Population IF		
		Preferred model	*K*_1_	*V*_ND_	*k*_3_	*K*_*i*_	*K*_*i*_ TRTV	Preferred model	*K*_*i*_	*K*_*i*_ TRTV	Preferred model	*K*_*i*_	*K*_*i*_ TRTV
S1	Test	2T3k	0.522	9.64	0.0016	0.0148	− 32%	2T3k	0.0118	6%	2T3k	0.0136	4%
	Retest	2T3k	0.420	8.02	0.0014	0.0108		2T3k	0.0126		2T3k	0.0142	
S2	Test	2T4k	0.577	10.12	0.0016	0.0157	− 23%	2T3k	0.0131	-3%	2T3k	0.0113	18%
	Retest	2T3k	0.407	5.90	0.0022	0.0125		2T3k	0.0127		2T3k	0.0136	
S3	Test	2T3k	0.341	7.33	0.0024	0.0166	− 9%	2T3k	0.0154	5%	2T3k	0.0193	4%
	Retest	2T4k	0.347	6.14	0.0026	0.0152		2T4k	0.0162		2T4k	0.0201	
S4	Test	2T3k	0.473	9.73	0.0025	0.0235	− 45%	2T3k	0.0202	− 17%	2T3k	0.0147	−24%
	Retest	2T3k	0.490	9.34	0.0016	0.0148		2T3k	0.0170		2T3k	0.0115	
S5	Test	2T3k	0.451	8.64	0.0025	0.0208	− 14%	2T3k	0.0200	− 5%	2T3k	0.0170	−9%
	Retest	2T3k	0.465	7.29	0.0026	0.0182		2T3k	0.0191		2T3k	0.0156	
Mean TRTV			13%	21%	20%	24.3% ± 14.5		7.3% ± 5.6			11.9% ± 8.9		
Mean test			0.473	9.09	0.0021	0.0183		0.0161			0.0152		
Mean retest			0.426	7.34	0.0021	0.0143		0.0155			0.0150		
		Preferred model	*K*_1_	*V*_ND_	*k*_3_	*K*_i_	% Δ*K*_*i*_	Preferred model	*K*_*i*_	% Δ*K*_*i*_	Preferred model	*K*_*i*_	% Δ*K*_*i*_
S6	Baseline	1T2k	0.528	9.78	0.0005	0.0046	182%	1T2k	0.0038	237%	2T4k	0.0071	113%
	Ketamine	2T3k	0.605	9.66	0.0014	0.0129		2T3k	0.0128		2T3k	0.0152	
S7	Baseline	2T4k	0.394	6.54	0.0029	0.0182	− 35%	2T4k	0.0170	0%	2T4k	0.0150	8%
	Ketamine	2T3k	0.487	9.75	0.0012	0.0118		2T3k	0.0169		2T4k	0.0162	
S8	Baseline	2T3k	0.683	11.72	0.0007	0.0083	1%	2T3k	0.0220	− 9%	2T3k	0.0183	−9%
	Ketamine	2T4k	0.559	10.30	0.0008	0.0084		2T3k	0.0201		2T3k	0.0167	
S9	Baseline	2T3k	0.368	7.74	0.0026	0.0193	− 36%	2T3k	0.0200	− 41%	2T3k	0.0114	−62%
	Ketamine	2T4k	0.328	6.56	0.0020	0.0124		2T3k	0.0118		2T3k	0.0044	
S10	Baseline	2T3k	0.488	10.51	0.0014	0.0143	30%	2T3k	0.0105	83%	1T2k	0.0011	−10%
	Ketamine	2T3k	0.600	9.84	0.0020	0.0186		2T3k	0.0192		1T2k	0.0010	
Mean baseline			0.492	9.25	0.0016	0.0129			0.0147			0.0106	
Mean ketamine			0.516	9.22	0.0015	0.0128			0.0162			0.0107	
% Change			5%	0%	−9%	−1%			10%			1%	

The unit of *K*_1_ is mL · cm^3^ · min^−1^ and the unit of *k*_3_ and *K*_*i*_ is min^−1^. *V*_ND_ equals *K*_1_/*k*_2_

*TRTV* test–retest variability, %Δ*K*_*i*_ the percentage change from the baseline scan

### Test–retest group

Mean TRT variability of whole brain GM *K*_*i*_ was 24% for scans analyzed using individual metabolite-corrected plasma input functions. A decrease in *K*_*i*_ was observed in retest scans for all subjects (range 8–37%). Across the nine ROIs, differences between mean test–retest *K*_*i*_ ranged from − 17% in thalamus to − 36% in brainstem. The Pearson correlation between scan interval in days and percentage difference in *K*_*i*_ was 0.99 (*p* = 0.002) for whole brain GM and 0.89 to 0.99 for individual ROIs. The TRT variability of *K*_1_, *k*_2_, *k*_3_, and *V*_ND_ did not correlate with scan interval. Furthermore, the correlation of *K*_*i*_ TRT variability with scan interval was not significant (*p* > 0.05) using within-subject and population-averaged input functions. Duration of daylight correlated moderately with *K*_*i*_ (*r* = 0.54, *p* = 0.11). ROI size showed a weak correlation with TRT variability (Spearman’s *ρ* = − 0.24, *p* = 0.11). The mean TRT variability was 13% for *K*_1_, 21% for *V*_ND_, and 20% for *k*_3_. TRT variability of GM *K*_*i*_ generated using the within-subject input functions was markedly improved at 7%. The single population-averaged input function returned a *K*_*i*_ TRT variability of 12%.

Analysis of the TACs (Fig. 2a–e) showed that mean GM SUV_AUC_ of test scans was 316 ± 40 SUV·min and of retest scans 307 ± 31 SUV·min. TRT variability of GM SUV_AUC_ was 13, 33, 4, 8, and 6% for subjects 1 to 5, respectively (mean TRT variability 12%). Whole brain SUV_90–120_ was, on average, 1.68 and 1.64 in test and retest scans (− 3% difference). Mean TRT variability of GM SUV_90–120_ was 12 ± 12%, and the between-subject coefficient of variation of SUV_AUC_ and SUV_90–120_ for all 10 PET1 scans (including baseline scans in the ketamine group) was 15 and 11%, respectively.

### Ketamine group

Ketamine administration led to whole brain GM *K*_*i*_ reductions in subjects 7 and 9, no change in subject 8, and increases in subjects 6 and 10 (data in Table 2). The effects of ketamine on *K*_*i*_ varied considerably between subjects, ranging from − 36 to 182%. Model fits were unstable across ROIs, and this was reflected in variable *K*_*i*_ changes between ROIs and subjects. The mean *V*_ND_ of whole brain GM did not change between baseline and ketamine scans. The mean rate of transport *K*_1_ in the baseline scans ranged from 0.33 to 0.68 between subjects. After ketamine, *K*_1_ increased in subjects 6, 7, and 10 by 15–24% and decreased in subjects 8 and 9 with 18 and 11%, respectively, resulting in an overall mean increase of 5%. Within-subject input functions led to an increase in the difference of *K*_*i*_ between the baseline and ketamine scans, ranging from − 41 to 237% in the five subjects. The population averaged input function did not improve the consistency in change of *K*_*i*_ either. Analysis of TACs (Fig. 2f–j) showed that whole brain SUV_AUC_ increased by 8, 27, 1, 1, and 17% following ketamine administration for subjects 6–10, respectively. Mean SUV_AUC_ of the baseline scans was 289 ± 51 SUV·min and of the ketamine scans 321 ± 66 SUV·min. The mean SUV_90–120_ of baseline and ketamine scans was 1.53 and 1.68, respectively (10% increase, owing to subjects 6, 7, and 10).

#### Plasma ketamine concentration

**Fig. 5:**
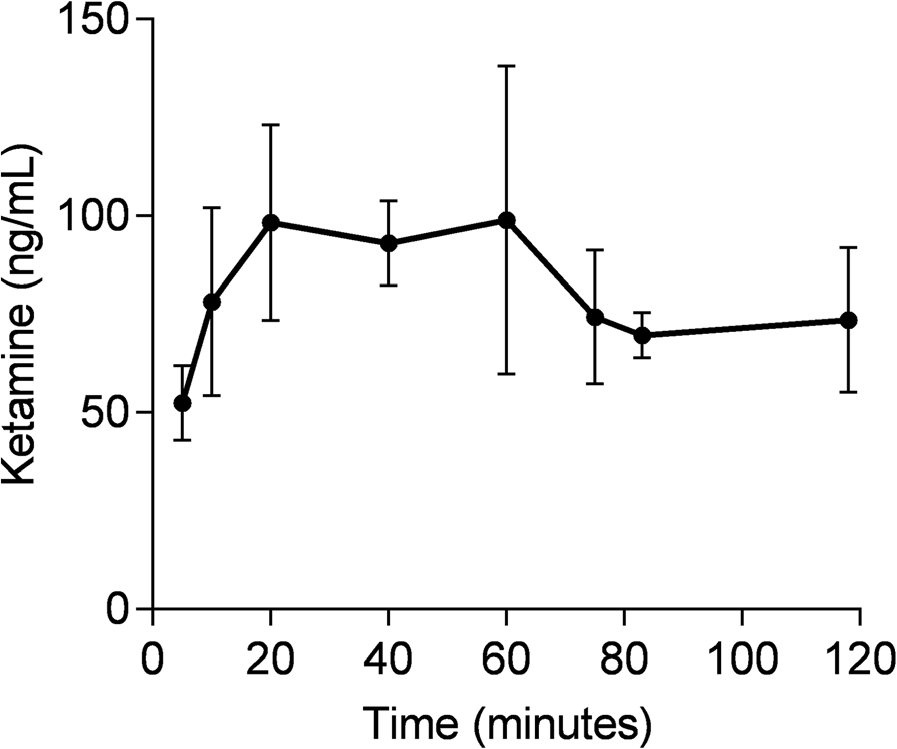
Mean plasma *S*-ketamine concentration during the course of the PET scan. Error bars represent standard deviations

Ketamine was well tolerated by all subjects. Feelings of dissociation were reported by all subjects at the start of PET scanning, but none showed loss of responsiveness or hypnosis. The plasma ketamine concentration could only be analyzed for three out of five subjects, and results are shown in Fig. 5.

## Discussion

Twenty PET scans were performed in 10 healthy subjects to evaluate [^18^F]PK-209 brain kinetics, test–retest reproducibility, and quantification of radiotracer binding to the intrachannel binding site of NMDA receptors. To this end, TRT variability was assessed in five subjects, followed by blocking experiments with intravenous 0.5 mg ∙ kg^−1^*S*-ketamine administration in five different subjects. The kinetic profile of [^18^F]PK-209 indicated irreversible binding, at least for the 120 min scan duration, suggesting a trapped binding mechanism or slow dissociation. The slow irreversible nature is in accordance with in vivo behavior of [^11^C]GMOM [10], the carbon-11 labeled analogue of PK-209. [^18^F]PK-209 readily entered the brain and displayed a fairly uniform pattern of uptake, with the rank-order of highest to lowest net influx constant (*K*_*i*_) in the volume-weighted ROIs being brainstem>cortex>cerebellum-thalamus-hippocampus-insula>dorsal striatum. There were no ROIs from which TACs were consistently favored by a kinetic model other than the 2T3k_V_B_ model. However, the significant correlation between 2T3k_V_B_ model agreement and ROI size indicated that TACs in smaller brain ROIs were affected more by noise and thus described adequately by several models. Previous kinetic analyses of PET scans with the structurally related radiotracer [^11^C]GMOM have shown similar heterogeneity in model preference between and within subjects [10].

### Test–retest variability

The TRT variability of [^18^F]PK-209 *K*_*i*_ calculated with single scan input functions was relatively large compared with reports of other radiotracers using similar equipment and techniques [21], at 24% in whole brain GM *K*_*i*_ and 17 to 36% in individual ROIs. A closer examination of SUV_AUC_ showed good consistency between test and retest scans, with a mean 12% difference. Tracer uptake in the last 30 min of the scan, SUV_90–120_, did not correlate with the irreversible rate of influx *K*_*i*_ in this group of subjects. Considering the small *k*_3_, it is likely that the PET signal at 90 to 120 min p.i. is still dominated by free and non-specific binding. Furthermore, the TACs themselves showed clear dissociation and washout of [^18^F]PK-209 during the course of the scan, suggesting that the ligand’s trapping component within the ion channel is relatively small. In the present study, a “coffee-break” protocol with extended scanning up to 4 h post radiotracer injection may have provided additional information on kinetics, such as the contribution of the ligand-binding site dissociation constant *k*_4_. However, tracer metabolism and low count rates at late timepoints would have complicated measurements of the input function.

Blood data from arterial measurements during the PET scan showed that there was rapid blood pool clearance combined with rapid tracer metabolism. Mean parent fractions were 57 and 34% at 10 and 20 min p.i., respectively, which is somewhat higher compared with non-human primate parent fractions [15]. Fast metabolism has also been shown in clinical PET studies of [^11^C]ketamine [22] and ligands from the class of bis(aryl)guanidines, i.e., [^11^C]CNS-5161 [23], [^11^C]GMOM [10], and [^18^F]GE-179 [24], which are structurally related to [^18^F]PK-209. HPLC analysis showed that one [^18^F]PK-209 metabolite accounted for approximately 30% of total plasma radioactivity from 20 min until the end of the scan. A limitation of the present study was that the fractional recovery of radioactivity from plasma declined over time, stabilizing at approximately 45%. Arterial samples taken immediately before PET were incubated with [^18^F]PK-209 at 37 °C, and radioactivity recovery of these samples remained constant at approximately 90% during the 2-h experiment. The difference between recovery fractions suggests that, during the scan, metabolites were formed in the body, which subsequently were retained within the pellet for corresponding samples. An influx of radiometabolites into the brain may have confounded [^18^F]PK-209 quantification and contributed to TRT variability. Ex vivo rodent studies have shown that polar metabolites only contribute 4% to total brain radioactivity at 60 min p.i. The non-polar metabolite fraction, however, was 18 and 25% at 15 and 60 min p.i., respectively [13]. Logan plots of [^18^F]PK-209 in non-human primates showed that linearity was attained from early to late timepoints, arguing against the significant accumulation of radiometabolites in the brain. Furthermore, recovery of radioactivity in the present study was high at the start of the scan, indicating that the analytical methods to measure parent [^18^F]PK-209 and associated input functions provided reliable results. TRT variability was further examined as a function of parent fraction determination in plasma. To this end, TACs were modeled with arterial input functions using parent fractions that were averaged within subjects and across the two samples of five subjects. Within-subject input functions led to a substantial reduction in *K*_*i*_ TRT variability from 24 to 7% and improved consistency of the 2T3k_V_B_ model preference from 14 to 17 (out of 20) PET scans.

Preliminary results showed a significant correlation between scan interval in days and reduction in *K*_*i*_ using the single-scan input function, but not with subject- and population-averaged input functions. This correlation is likely due to either a systemic error in test or retest input function measurements or a true biological component resulting in lowered availability of the NMDA channel site for radiotracer binding during the second scan. In same-day test–retest studies with the metabotropic glutamatergic tracers [^11^C]ABP688 and [^18^F]FPEB, it was shown that binding was significantly higher in the second scan, whereas two scans days to weeks apart showed good TRT variability [25]. Diurnal and seasonal variation in receptor or endogenous ligand concentrations might be sources of increased TRT variability. In the present study, however, all scans were performed in the afternoon between 12:00 and 15:00 h, limiting diurnal effects on glutamatergic neurotransmission. The moderate correlation of *K*_*i*_ with duration of daylight and strong correlation of *K*_*i*_ with scan interval could be indicative of seasonal effects. These have been found previously in serotonin 5-HT_1A_ receptor PET studies [26]. Finally, and taking into consideration the limitations of a small sample size, it is noteworthy that the two subjects aged 34 and 37 showed numerically higher TRT variability compared with the three subjects aged 22–23. Future work is needed to understand the source of variability and a full validation of these findings will require a larger cohort.

### Ketamine blocking studies

Despite good quality data for all [^18^F]PK-209 scans, there was no consistent effect of ketamine administration on the pharmacokinetic model parameters. Intravenous ketamine administration increased whole brain SUV_AUC_ and SUV_90–120_ in three out of five subjects, whereas previous non-human primate experiments showed a 15% reduction in mean SUV_AUC_ after MK-801 administration [15]. The best PET model fits changed in four out of five subjects following ketamine administration, but not in a predictable manner. The unstable fits of [^18^F]PK-209 may be explained by changes in the arterial input function or brain pharmacokinetics of the tracer. For example, the increased SUV in subjects 6, 7, and 10, which is reflected in increased *K*_*1*_ values, shows that the uptake of [^18^F]PK-209 is blood flow-dependent. Ketamine is known to exert direct vasodilatory effects on the cerebral vasculature through a calcium-dependent mechanism. In a review of 20 human imaging studies, it was shown that plasma ketamine concentrations, comparable to those used in the present study, increased global and/or regional cerebral blood flow in human subjects [27]. Contrasting results from a recent simultaneous PET and functional MRI imaging study in anesthetized non-human primates demonstrated that cerebral blood volume following administration of the PCP site blocker GE-179 was acutely reduced [28]. In a bolus-plus-infusion paradigm, “cold” GE-179 at a dose of 0.6 mg ∙ kg^−1^ was administered when [^18^F]GE-179 was at steady state, which was expected to reduce the brain signal by competitive displacement at the PCP binding site. A short-term blood volume decrease was observed; however, GE-179 did not significantly block the PET signal and had no effect on arterial plasma blood levels, indicating that the [^18^F]GE-179 signal is independent of flow [28]. Ketamine may affect blood flow differently than GE-179, and a future study in humans with simultaneous [^18^F]PK-209 PET and fMRI could elucidate the relationship between NMDA-R blockers, blood flow, and radiotracer binding.

The arterial plasma fractions of [^18^F]PK-209 in the baseline scans were on average 8 to 15% lower than in the ketamine scans (Fig. 4), but also 16% lower compared with the TRT group. The small increase in baseline metabolism of subjects 6 to 10 may suggest an initial group difference which was normalized by ketamine administration. However, in non-human primates, there was no effect of MK-801 on [^18^F]PK-209 metabolism, nor did ketamine administration affect [^11^C]GMOM metabolism in humans. Systematic errors in the estimation of arterial blood parameters or natural variability in metabolism may underlie the observed differences. Although the measurement error may have affected the model parameters, it is unlikely to explain the variability of [^18^F]PK-209 *K*_*i*_ between subjects following ketamine administration.

The mean plasma ketamine concentrations plateaued at ~ 100 ng ∙ mL^−1^ between 20 and 60 min after the start of PET (40 min since the start of ketamine infusion) and decreased to ~ 70 ng ∙ mL^−1^ at the end of the scan, 160 min since the start of 0.5 mg ∙ kg^−1^ ketamine infusion. As expected, the *C*_max_ in the current study was dose-proportionally higher than the 0.3 mg ∙ kg^−1^ ketamine administered in the [^11^C]GMOM blocking study [10]. Subjects in a SPECT study by Stone et al. [29] were administered 1.1 mg ∙ kg^−1^*S*-ketamine over 75 min, which led to a mean plasma concentration of 173 ng ∙ mL^−1^ and displacement of the NMDA-R channel ligand [^123^I]CNS-1261 in the brain. Preclinical in vivo studies have also demonstrated a strong and rapid temporal relationship between ketamine concentrations in plasma and radiotracer inhibition in brain tissue. For example, in rats, 67% inhibition of [^3^H]MK-801 binding was observed at 1 min post-dosing with 3 mg ∙ kg^−1^ racemic (±)ketamine IV, and the inhibition declined to 19% at 20 min post-dose [30]. The plasma racemic ketamine concentration required to inhibit 50% of specific [^3^H]MK-801 binding in vivo has been calculated in the range of 1.9–3.7 μM [30]. The present pharmacokinetic data show a concentration of ketamine in plasma of 0.3–0.4 μM during PET scanning, which may have been insufficient to unmask specific uptake of [^18^F]PK-209. Nevertheless, *S*-ketamine at a lower plasma concentration of 0.26 μM was shown to reduce the *K*_*i*_ of the equipotent carbon-11 labeled analogue of PK-209, [^11^C]GMOM, in humans [10]. The concentrations of unlabelled PK-209 and GMOM that inhibit specific binding of [^3^H]MK-801 to rat forebrain membranes were shown to be similar at 18.4 and 21.7 nM respectively [13]. Furthermore, data from the preclinical [^18^F]PK-209 PET study demonstrated that a dose of 0.3 mg ∙ kg^−1^ MK-801 reduced the volume of distribution in two out of three rhesus monkeys compared with baseline [15]. In displacement binding studies, MK-801 is two orders of magnitude more potent than (±)ketamine at the ion channel site [30], but the compound is not approved for human use and therefore could not be implemented in the present study design. Despite these reports of inhibition of NMDA-R activity by channel blockers, the recent preclinical in vivo evaluation of [^18^F]GE-179 suggests that the PET signal is largely non-specific [28].

NMDA receptors are complex, highly modulated ligand-gated ion channels bound in cell membranes that, in order to open, require activation of nearby AMPA and kainate receptors as well as co-activation by glutamate and d-serine or glycine. Recently, electron cryomicroscopy experiments revealed how small molecules affect the NMDA-R structure and ion channel opening [31]. Many endogenous ligands acting at NMDA-Rs, such as Mg^2+^, Zn^2+^, H^+^, polyamine cations, neurosteroids, and fatty acids, determine the in vivo binding properties of ligands targeted for NMDA receptors. For example, it has been shown that in native NMDA receptors of rat hippocampus CA1 pyramidal neurons, IC_50_ values of NMDA-R channel blockers are increased 1.5 to 5 times compared with magnesium-free conditions [32]. Variations in physiological Mg^2+^ or other endogenous ligands could have affected [^18^F]PK-209 binding and may have contributed to the observed TRT variability and inconsistent ketamine effects. A second possibility is that ketamine and [^18^F]PK-209 inhibit distinct populations of NMDA-Rs. Ketamine predominantly inhibits synaptic NMDA-Rs, whereas for example memantine primarily inhibits extrasynaptic NMDA-Rs [33], although more weakly [34]. In this respect, it may be valuable to investigate memantine as a pharmacological blocker in future PET studies with NMDA-R radiotracers to examine different domains of [^18^F]PK-209 binding. A third possibility is that ion channel ligands exhibit biexponential association kinetics with the NMDA ionophore and thereby complicate PET pharmacokinetic modeling. Very few studies have examined how the association rate constants of ion channel blockers change as a function of radioligand concentration, and there is evidence to suggest that the kinetics of channel blocker association with the NMDA ionophore do not follow the law of mass-action [2]. Changing the ligand-binding site accessibility can change the rate of association and dissociation, but has no effect on equilibrium affinity of ligand binding [35]. One cannot exclude that the slightly different doses of [^18^F]PK209 injected in the baseline versus the blocking scans may have contributed to a noisy data set.

## Conclusions

The establishment of a radiotracer for in vivo quantification of specific binding to the NMDA receptor remains challenging. The divergent clinical and preclinical behavior of [^18^F]PK-209 could be due to multiple differences in interactions between exogenous and endogenous glutamatergic ligands. There are plausible biophysical explanations that remain to be tested, which are pivotal for interpreting the ligand-NMDA-R interactions. The conclusion of the present study is that the molecular interaction between [^18^F]PK-209 and the binding site within the NMDA-R ion channel was not shown to be reproducible or specific. It is possible that the typical study design for neuroreceptor PET tracers executed here is not applicable to such a dynamic and complex receptor system as the NMDA-R.

## Abbreviations

2T3k: Two-tissue irreversible compartmental model
3-D RAMLA: 3-Dimensional row action maximum likelihood reconstruction algorithm
AIC: Akaike Information Criterion
AMPA: α-Amino-3-hydroxy-5-methyl-4-isoxazolepropionate
AUC: Area under the curve
GM: Gray matter
*K*_*i*_: Inhibition constant
MRI: Magnetic resonance imaging
NMDA-R: *N*-methyl-d-aspartate receptor
P.i.: Post injection
PCP: Phencyclidine
PET: Positron emission tomography
ROI: Region of interest
SD: Standard deviation
SPECT: Single-photon emission computed tomography
SUV: Standardized uptake value
TAC: Time-activity curve
TRT: Test–retest
V_ND_: Volume of distribution

## References

[1] Paoletti P, Bellone C, Zhou Q (2013). NMDA receptor subunit diversity: impact on receptor properties, synaptic plasticity and disease. Nat Rev Neurosci.

[2] Javitt DC, Zukin SR (1989). Biexponential kinetics of [3H]MK-801 binding: evidence for access to closed and open N-methyl-D-aspartate receptor channels. Mol Pharmacol.

[3] Traynelis SF, Wollmuth LP, McBain CJ (2010). Glutamate receptor ion channels: structure, regulation, and function. Pharmacol Rev.

[4] Lee C-H, Lü W, Michel JC (2014). NMDA receptor structures reveal subunit arrangement and pore architecture. Nature.

[5] Danysz W, Parsons CG (2012). Alzheimer’s disease, β-amyloid, glutamate, NMDA receptors and memantine--searching for the connections. Br J Pharmacol.

[6] Ametamey SM, Bruehlmeier M, Kneifel S (2002). PET studies of 18F-memantine in healthy volunteers. Nucl Med Biol.

[7] Asselin M, Hammers A, Turton D, Osman S, Koepp M, Brooks D (2004). Initial kinetic analyses of the in vivo binding of the putative NMDA receptor ligand [C-11]CNS 5161 in humans. Neuroimage.

[8] Hartvig P, Valtysson J, Lindner KJ (1995). Central nervous system effects of subdissociative doses of (S)-ketamine are related to plasma and brain concentrations measured with positron emission tomography in healthy volunteers. Clin Pharmacol Ther.

[9] Klein PJ, Chomet M, Metaxas A (2016). Radiolabeling and evaluation of novel amine guanidine derivatives as potential positron emission tomography tracers for the ion channel of the *N* -methyl-d-aspartate receptor. Eur J Med Chem.

[10] van der Doef TF, Golla SS, Klein PJ, et al. Quantification of the novel *N*-methyl-d-aspartate receptor ligand [11C]GMOM in man. J Cereb Blood Flow Metab. 2016;36:1111–21. 10.1177/0271678X15608391 PMC4904354 26661185

[11] Waterhouse RN, Slifstein M, Dumont F (2004). In vivo evaluation of [11C]*N*-(2-chloro-5-thiomethylphenyl)-*N*′-(3-methoxy-phenyl)-*N*′-methylguanidine ([11C]GMOM) as a potential PET radiotracer for the PCP/NMDA receptor. Nucl Med Biol.

[12] van der Aart J, van der Doef TF, Horstman P (2017). Human dosimetry of the *N*-methyl-d-aspartate receptor ligand ^11^ C-GMOM. J Nucl Med.

[13] Klein PJ, Christiaans JAM, Metaxas A (2015). Synthesis, structure activity relationship, radiolabeling and preclinical evaluation of high affinity ligands for the ion channel of the *N*-methyl-d-aspartate receptor as potential imaging probes for positron emission tomography. Bioorg Med Chem.

[14] Klein PJ, Schuit RC, Metaxas A (2017). Synthesis, radiolabeling and preclinical evaluation of a [11C]GMOM derivative as PET radiotracer for the ion channel of the *N*-methyl-d-aspartate receptor. Nucl Med Biol.

[15] Golla SSV, Klein PJ, Bakker J (2015). Preclinical evaluation of [18F]PK-209, a new PET ligand for imaging the ion-channel site of NMDA receptors. Nucl Med Biol.

[16] Surti S, Kuhn A, Werner ME (2007). Performance of Philips Gemini TF PET/CT scanner with special consideration for its time-of-flight imaging capabilities. J Nucl Med.

[17] Vollmar S, Michel C, Treffert JT (2002). HeinzelCluster: accelerated reconstruction for FORE and OSEM3D. Phys Med Biol.

[18] Zhang Y, Brady M, Smith S (2001). Segmentation of brain MR images through a hidden Markov random field model and the expectation-maximization algorithm. IEEE Trans Med Imaging.

[19] Hammers A, Allom R, Koepp MJ (2003). Three-dimensional maximum probability atlas of the human brain, with particular reference to the temporal lobe. Hum Brain Mapp.

[20] Akaike H (1992). Data analysis by statistical models. No To Hatatsu.

[21] van Assema DM, Lubberink M, Boellaard R (2012). Reproducibility of quantitative (R)-[11C]verapamil studies. EJNMMI Res.

[22] Kumlien E, Hartvig P, Valind S (1999). NMDA-receptor activity visualized with (*S*)-[*N*-methyl-11C]ketamine and positron emission tomography in patients with medial temporal lobe epilepsy. Epilepsia.

[23] Schiffer WK, Pareto-Onghena D, Wu H (2005). In vivo evaluation of [11C]CNS-5161 as a use-dependent ligand for the NMDA glutamate receptor channel. J Cereb Blood Flow Metab.

[24] McGinnity CJ, Hammers A, Riaño Barros DA (2014). Initial evaluation of 18F-GE-179, a putative PET tracer for activated *N*-methyl d-aspartate receptors. J Nucl Med.

[25] DeLorenzo C, Gallezot JD, Gardus J (2017). In vivo variation in same-day estimates of metabotropic glutamate receptor subtype 5 binding using [11C]ABP688 and [18F]FPEB. J Cereb Blood Flow Metab.

[26] Matheson GJ, Schain M, Almeida R (2015). Diurnal and seasonal variation of the brain serotonin system in healthy male subjects. Neuroimage.

[27] Zeiler FA, Sader N, Gillman LM (2016). The cerebrovascular response to ketamine: a systematic review of the animal and human literature. J Neurosurg Anesthesiol.

[28] Schoenberger M, Schroeder FA, Placzek MS (2018). In vivo [ 18 F]GE-179 brain signal does not show NMDA-specific modulation with drug challenges in rodents and nonhuman primates. ACS Chem Neurosci.

[29] Stone JM, Erlandsson K, Arstad E (2008). Relationship between ketamine-induced psychotic symptoms and NMDA receptor occupancy—a [123I]CNS-1261 SPET study. Psychopharmacology.

[30] Fernandes A, Wojcik T, Baireddy P (2015). Inhibition of in vivo [3H]MK-801 binding by NMDA receptor open channel blockers and GluN2B antagonists in rats and mice. Eur J Pharmacol.

[31] Zhu S, Stein RA, Yoshioka C (2016). Mechanism of NMDA receptor inhibition and activation. Cell.

[32] Nikolaev MV, Magazanik LG, Tikhonov DB (2012). Influence of external magnesium ions on the NMDA receptor channel block by different types of organic cations. Neuropharmacology.

[33] Johnson JW, Glasgow NG, Povysheva NV (2015). Recent insights into the mode of action of memantine and ketamine. Curr Opin Pharmacol.

[34] Song X, Jensen MØ, Jogini V (2018). Mechanism of NMDA receptor channel block by MK-801 and memantine. Nature.

[35] Starmer CF, Packer DL, Grant AO (1987). Ligand binding to transiently accessible sites: mechanisms for varying apparent binding rates. J Theor Biol.

